# Quantitative Sensory Testing in Painful Hand Osteoarthritis Demonstrates Features of Peripheral Sensitisation

**DOI:** 10.1155/2012/703138

**Published:** 2012-11-14

**Authors:** Julekha Wajed, Vivian Ejindu, Christine Heron, Monika Hermansson, Patrick Kiely, Nidhi Sofat

**Affiliations:** ^1^Department of Biomedical Sciences, St. George's University of London, Cranmer Terrace, London SW17 ORE, UK; ^2^Department of Radiology, St. George's Hospital, London SW17 OQT, UK

## Abstract

Hand osteoarthritis (HOA) is a prevalent condition for which treatments are based on analgesia and physical therapies. Our primary objective was to evaluate pain perception in participants with HOA by assessing the characteristics of nodal involvement, pain threshold in each hand joint, and radiological severity. We hypothesised that inflammation in hand osteoarthritis joints enhances sensitivity and firing of peripheral nociceptors, thereby causing chronic pain. Participants with proximal and distal interphalangeal (PIP and DIP) joint HOA and non-OA controls were recruited. Clinical parameters of joint involvement were measured including clinical nodes, VAS (visual analogue score) for pain (0–100 mm scale), HAQ (health assessment questionnaire), and Kellgren-Lawrence scores for radiological severity and pain threshold measurement were performed. The mean VAS in HOA participants was 59.3 mm ± 8.19 compared with 4.0 mm ± 1.89 in the control group (*P* < 0.0001). Quantitative sensory testing (QST) demonstrated lower pain thresholds in DIP/PIP joints and other subgroups in the OA group including the thumb, metacarpophalangeal (MCPs), joints, and wrists (*P* < 0.008) but not in controls (*P* = 0.348). Our data demonstrate that HOA subjects are sensitised to pain due to increased firing of peripheral nociceptors. Future work to evaluate mechanisms of peripheral sensitisation warrants further investigation.

## 1. Introduction

Osteoarthritis (OA) is the commonest form of arthritis worldwide, affecting increasing numbers of people in an ageing population [1]. Among US adults, nearly 27 million people have osteoarthritis [2]. The US Framingham study found that 27% of adults aged over 26 have hand OA (HOA) [2]. In a European study of 7983 people, 25% of participants with hand pain showed significant hand disability [3]. Chronic pain, particularly in the functional context of the hand, causes significant emotional and financial burden to those affected, impacting on carers and on society as a whole. Treatment of HOA currently comprises analgesia with paracetamol, nonsteroidal anti-inflammatory drugs (NSAIDs), opioid analgesics, and rehabilitative hand physiotherapy [4]. However, large numbers of people continue to experience impaired hand function and pain. There is therefore a pressing unmet need to improve management of HOA. 

Recent work has focused on aiming to understand mechanisms of pain in OA [5]. Previous studies have shown subchondral inflammation and neoinnervation at the local joint level, which is accompanied by vascular invasion in the subchondral plate in the OA joint [6]. In addition, modification of neural networks may occur during OA, which reflects long-term changes in expression of neurotransmitters, their receptors, and neural ion channels. These processes contribute to altered pain perception, also known as sensitisation [5]. Although several well-validated pain outcome measures are in use in clinical practice, for example, the Western Ontario and McMaster Universities Arthritis Index (WOMAC) and visual analogue score for pain (VAS), a major issue in OA is how to quantify pain objectively in patients. Several groups have recently reported the use of quantitative sensory testing (QST) [7, 8]. Pain threshold testing using algometers has become more widely accepted for measuring pain perception objectively since it demonstrates low variability over time in people with knee OA [7] or intraoral pain [8]. Recently, Suokas et al. published a meta-analysis showing that quantitative sensory testing of pain pressure thresholds demonstrated good reproducibility in differentiating between people with OA and healthy controls [9]. In their meta-analysis, Suokas et al. found that pain pressure threshold testing in OA-affected sites suggested peripheral sensitisation and central sensitisation in remote sites [9].

The primary aim of our study was to evaluate pain perception in a cohort of participants with hand OA by assessing the characteristics of nodal involvement, pain thresholds in both hands, and radiological severity. The secondary aim of our study was to evaluate any features of sensitisation in our cohort of subjects with hand OA compared with healthy non-OA controls.

## 2. Methods

### 2.1. Participants

The London-Surrey Borders Research Ethics Committee provided ethical approval for this study, reference 09/H0718/60. Thirteen participants with hand OA were recruited from rheumatology outpatient clinics at St George's Hospital, London. All participants had hand pain due to primary OA of DIP (distal interphalangeal joints) and PIP (proximal interphalangeal) joints. Inclusion criteria were age range 40–85, female gender, and fulfilling ACR clinical criteria for HOA [10]. Exclusion criteria included another rheumatological diagnosis, for example, rheumatoid arthritis, recent surgery, male gender, diabetes mellitus, psychiatric disorders, and other neurologic conditions. Secondary OA (posttraumatic, metabolic, and inflammatory rheumatic disease) patients were also excluded. Healthy controls were recruited through poster advertisements at St George's Hospital and St George's University of London. The absence of hand OA was checked in this population by a clinical history and examination by two qualified rheumatologists (J. Wajed/N. Sofat). The same exclusion criteria as for the OA group were applied. The controls were matched for gender, age range, no other comorbidities including ischaemic heart disease, psychiatric disorders, and diabetes mellitus. Thirteen female controls were recruited through poster advertisements. This study was limited to females due to recognised gender differences in pain perception [11, 12]. A homogeneous cohort of female HOA participants and controls was therefore recruited.

### 2.2. Clinical Evaluation

**Figure 1:**
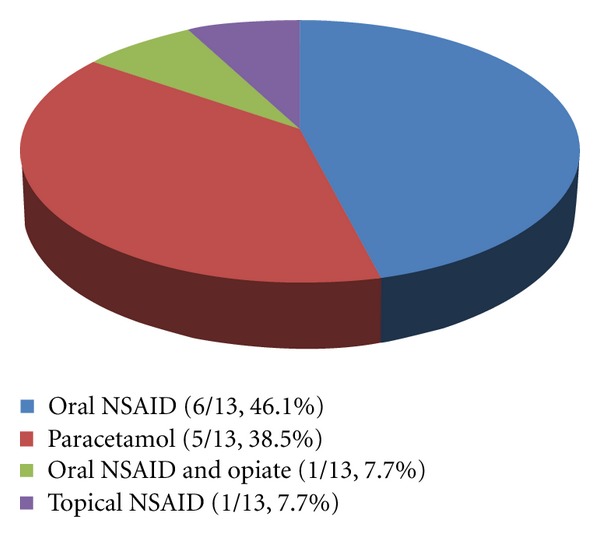
Hand OA participant analgesic use. The subgroups with respect to analgesic use in the hand OA group were classified as (1) oral NSAID including diclofenac, ibuprofen, or celecoxib; (2) paracetamol up to 1 g four times daily; (3) oral NSAID (diclofenac) plus opiate (cocodamol); (4) topical NSAID (ibuleve gel). Proportions of participants in each group are shown in addition to percentages.

A number of self-administered questionnaires were completed including the Visual Analogue Score (VAS) for pain (0–100 mm) [13], for which participants were asked their average pain score in their hands in the previous week, the Health Assessment Questionnaire (HAQ) [14], and the Hospital Anxiety and Depression Scale (HADs) [15]. Use of analgesic drugs by the HOA group and controls was recorded (Figure 1). All HOA participants underwent plain radiography of both hands using standardised views. Kellgren-Lawrence scores (K-L) [16] were agreed by consensus opinion of two senior musculoskeletal radiologists (V. Ejindu and C. Heron). A radiographic training atlas for OA [17] was used and before the scoring, each consultant radiologist also underwent a training session. K-L scores for each individual hand joint: thumb carpometacarpal (CMC), metacarpophalangeal (MCP), and interphalangeal (IP) joints and all MCPs, PIPs, and DIPs) were recorded at each joint specified in the range 0–4 using standard criteria [17]. This allowed K-L scores for individual joints to be related to algometer scores for each joint measured. A hand-held digital algometer (Wagner Instruments, CT, USA) was used to test pain threshold in all participants in both hands with *n* = 30 regions for each participant, 780 regions in total. Regions tested included dorsal aspects of all DIP, PIP, and MCP joints of each digit and thumb and the dorsum of each wrist. The 1 cm^2^ flat rubber algometer probe was held perpendicular to the dorsal aspect of the skin and force was applied to provide a constant increase in pressure at a rate of 1 Newton/cm^2^ per sec. Therefore, the algometer scores are stated as N/cm^2^ in all reported results. The individual was asked to say “stop” when the sensation of pressure became the first sensation of pain. The algometer was applied by each joint being examined 3 times in succession with an interval between applications. After all three readings were taken, the average from the last 2 readings was calculated as the pain pressure threshold. The intervals between each algometer measurement were long enough to prohibit temporal summation (TS). Temporal summation occurs when a series of nerve impulses arrives at a synapse so that the duration of the impulses is shorter than the postsynaptic potential, thereby meaning that the delivery of transmitted impulses is combined to create a larger than normal impulse [18]. This phenomenon has also been described as temporal summation of “second pain” (TSSP) or “windup,” which results from the summation of C-fibre-evoked responses of dorsal horn neurons [19]. The QST involved taking 3 readings from 15 joints in both hands, that is, 45 readings from a relatively small anatomical area. Since the pain pressure thresholds in the joints being tested were in close proximity, the QST protocol was designed, so that pressure was applied over the bony surfaces of the dorsal aspects of the joints affected. In addition, where the joints were in very close proximity, that is, the knuckles, typically the following order was taken when measuring pain thresholds: 1st CMC, 3rd MCP, 5th MCP, 2nd MCP, 4th MCP, and so on. Previous studies have suggested an induction of temporal summation at 0.33 Hz in participants with musculoskeletal pain [19]. In our study, we allowed a variable pause between 1 and 5 seconds between each application of pain pressure threshold testing in order to avoid effects of temporal summation in our HOA and control groups. 

### 2.3. Statistics

All clinical data was analysed using Graphpad Prism (CA, USA). Data was expressed as mean ± standard deviation unless otherwise stated. For correlation analyses, Spearman rank correlations were performed with a 95% confidence interval and two-tailed *P* values. Assessment of agreement of kappa scores between the two consultant radiologists was performed using an online kappa calculator http://justusrandolph.net/kappa/ and has been well-validated [20] in previous studies. For comparing group means, the Mann-Whitney *U* statistical test for comparison of nonparametric data was used. For comparing if values in pain thresholds varied between different subgroups, the Kruskal-Wallis test was used. Significance was considered to be at *P* ≤ 0.05 in all analyses.

## 3. Results

### 3.1. Hand OA Participants Have Higher VAS Scores and Significant Functional Impairment Compared to Controls

For the HOA group, mean age was 61.0 ± 3.79, and the control group was slightly younger with a mean age 52.8 ± 1.47, with no statistically significant difference in age between the two groups (*P* = 0.12) (Table 1). All hand OA participants had a family history of OA with involvement at other sites including the knee, hip, shoulder, ankle, and spine (Table 1). The two groups were well matched with respect to comorbidities, which included hypertension, hypothyroidism, carcinoma of the breast, dyspepsia and intervertebral disc disease. Only 1 participant in the hand OA group had previous joint replacement surgery of the hip.

All participants were female, with a mean duration of diagnosis for the hand OA group of 6.1 years (SD 1.19) (Table 2). VAS, HAQ, and HADS scores were significantly different between groups (Table 2). The HOA group reported significantly high pain scores with a mean VAS of 59.3 ± 8.19 compared with controls of 4.0 ± 1.89 (*P* < 0.0001). The HOA group demonstrated significant functional impairment, with HAQ scores 8-fold higher than controls (*P* < 0.0001). When considering HADS scores for anxiety and depression, HOA participants had significantly higher values on anxiety scales (*P* < 0.01) and depression scales (*P* < 0.05) than controls. This finding suggests that HOA subjects may anticipate higher anxiety while considering performing functional tasks with their hands. It is also notable that although the HOA participants did report higher anxiety levels, they did not reach clinical diagnostic levels of anxiety and depression. From Tables 1 and 2, one can observe that the control group is slightly younger, differing by an average of 8 years. Due to such age differences, it is possible that age could have had an effect on reporting of anxiety and depression and pain thresholds. It was also observed that the hand OA and healthy control groups were all right-handed and had similar occupational demographics with predominantly sedentary occupations (Table 3). In the hand OA group all subjects had symptoms of pain in both hands, suggesting that lifting or carrying heavy objects, or handedness, was not involved in the development of their symptoms. There was a much higher preponderance for a family history of OA in the hand OA group than the healthy controls with similar levels of comorbidity in both groups. 

All hand OA participants were taking analgesics, with 92% on oral analgesic drugs (Figure 1). Figure 1 illustrates the variety of prescribed analgesics utilised by the hand OA cohort including paracetamol use alone (5/13, 38.5%), oral NSAID use (including ibuprofen, diclofenac, and celecoxib) (6/13, 46.1%), oral NSAID and opiate use (1/13, 7.7%), and topical NSAID use (1/13, 7.7%). No controls were taking regular analgesic medication. Of the hand OA participants, many had severe symptomatic complaints reflected by the observation that 92% required oral analgesic drugs. Of the hand OA group, 69.2% had involvement in other joints including the spine, knee, hip, and ankles.

### 3.2. Pain Thresholds Are Reduced in Hand Joints of OA Participants Compared with Controls

**Figure 2:**
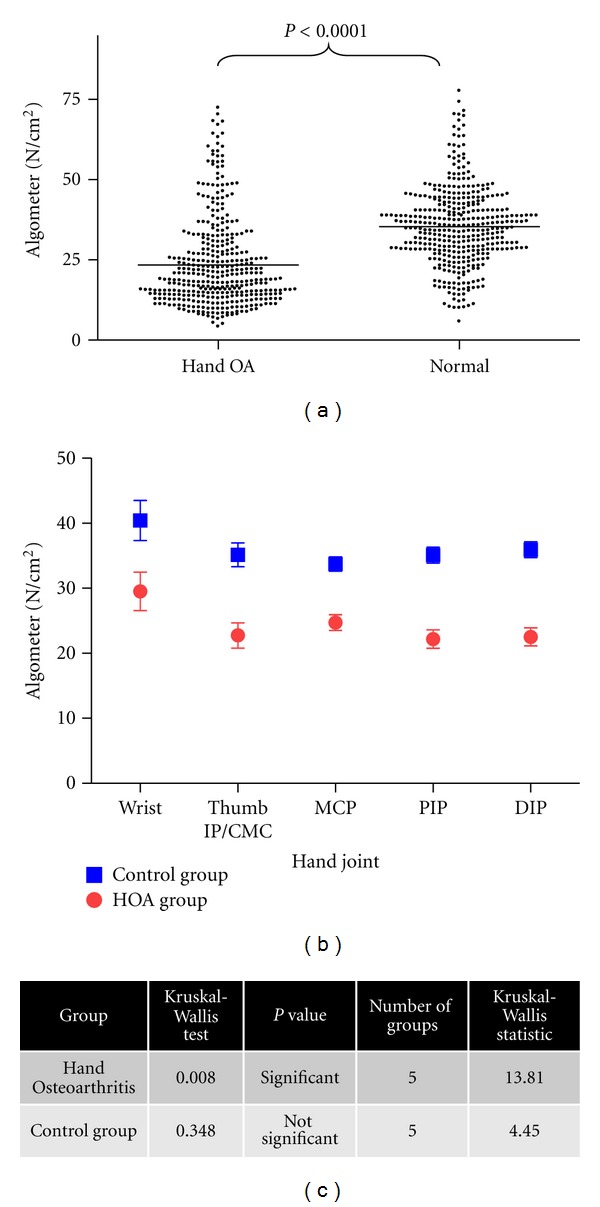
Pain threshold analysis in hand OA and control group. (a) Plot of algometer scores in Newtons per square cm (N/cm^2^) for a total of 780 joints in the hand OA participants and controls is shown. Each point represents one hand joint region algometer score with mean values indicated by the solid line. (b) Subgroup analysis of data in hand OA and control groups stratified according to joint region involved. The 5 subgroups include wrist, thumb 1st CMC/IP, MCPs, PIPs, and DIPs. Data plotted are mean ± SEM. SEM was used to account for the varying number of joints between different groups (c). Statistical comparison between groups is shown in (b). The Kruskal-Wallis test was used to evaluate statistical significance between the subgroups within the hand OA and control groups. A significant difference between groups was considered at *P* ≤ 0.05.

The evaluation of pain thresholds in our study demonstrated that HOA participants showed significantly lower algometer scores (mean 23.5, SD 11.9 Newtons) than controls (mean 34.1, SD 13.8 N/cm^2^, *P* < 0.0001) (Figure 2(a)). In a subgroup analysis of mean pain thresholds in the wrist, thumb 1st CMC/IP, MCPs, PIPs, and DIPs, Figures 2(b) and 2(c) show that pain thresholds did not vary significantly between different subgroups in the control group (*P* = 0.34). In contrast, there was a statistically significant difference in subgroups between the subgroups assessed in the hand OA group, with pain thresholds in the thumb 1st CMC/IP, MCPs, PIPs, and DIPs varying significantly with the wrist (*P* = 0.008).

**Figure 3:**
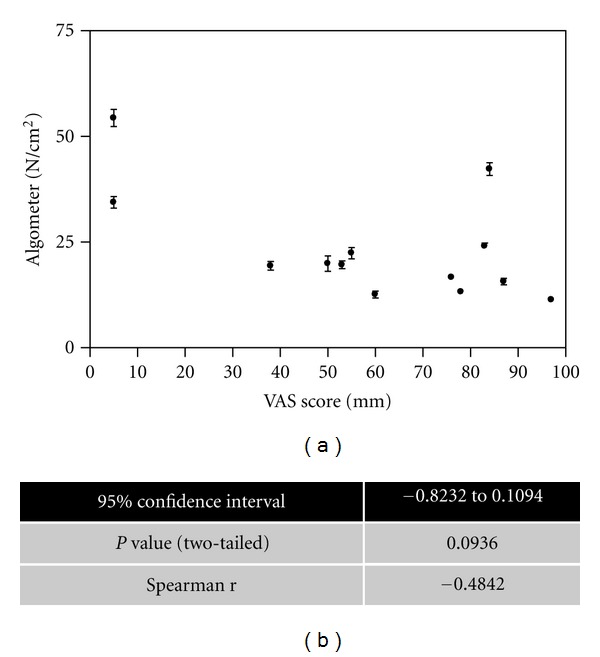
Correlation analysis between VAS and algometer scores. A correlation plot for hand OA participants between VAS scores (mean ± SD) and algometer scores is shown. Spearman correlation analysis for statistical significance is shown. A significant difference between groups was considered at *P* ≤ 0.05.

**Figure 4:**
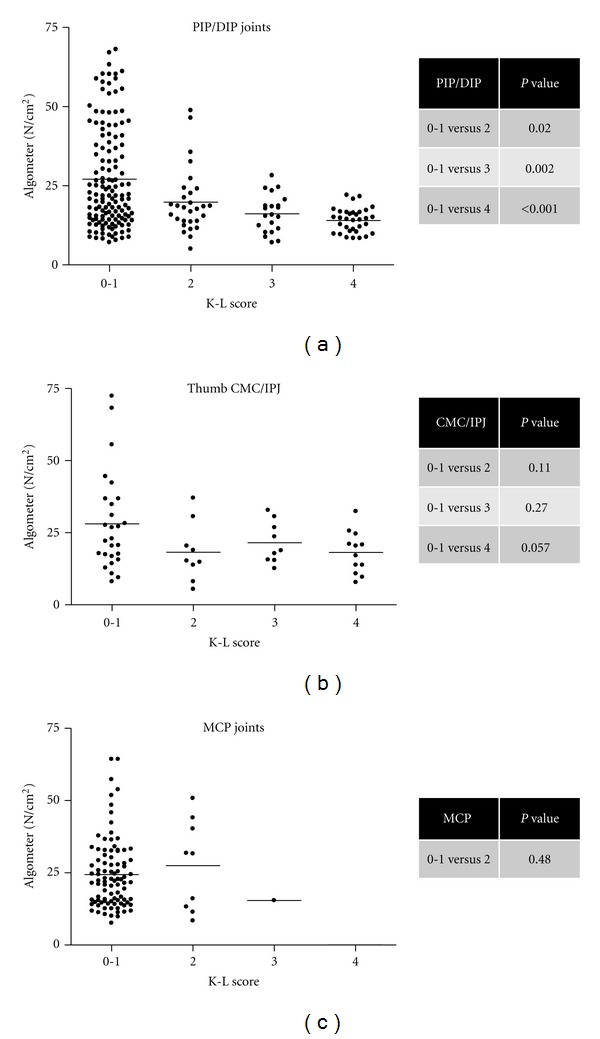
Comparison of pain pressure thresholds by algometer testing with Kellgren-Lawrence score by joint distribution in the hand OA cohort. Algometer versus K-L scores are also shown in DIP/PIP joints (a), thumb IP joints (b), and MCP joints (c). Differences between groups were analysed using an unpaired *t*-test (significance considered at *P* ≤ 0.05).

We next evaluated the relation between clinical nodes, radiographic grade, and pain thresholds. The proportion of palpable nodes in the HOA cohort was 49% (191/390), all of which were in the PIP/DIP joints. Of all the joints assessed, the percentage of hand joints with K-L grade ≥ 2 was 49.2% (192/390). There was no significant correlation between pain thresholds and participant-reported VAS scores in the hand OA group (*P* = 0.094, *r* value = −0.484) (Figure 3), indicating that VAS scores and pain thresholds may be testing distinct components of pain perception. In the joints most severely affected both clinically and radiographically, that is, the PIP and DIP joints, increasing radiological severity was associated with a significantly lower pain threshold (Figure 4). The same relationship was not observed in the thumb 1st CMC/IP or MCP joints in the hand OA group (Figure 4). In the wrist, the proportion of subjects with K-L score 0–2 was 21/26 and K-L score 3-4 was 5/26. Data in Figure 2(b) demonstrate that the mean pain thresholds were higher in the wrist than other subgroups in the hand OA group (mean 29.5 ± SEM 3.0), but that the hand OA group had a lower mean pain threshold at the wrist than the control wrist group (mean 40.4 ± SEM 3.1).

The agreement between observers (two consultant radiologists VE and CH) for the Kellgren-Lawrence grading was calculated using a kappa score calculation. This showed that the percent overall agreement was 0.769 with a fixed marginal kappa of 0.257 and a free marginal kappa of 0.538, suggesting good agreement of K-L grading between the two observers [20].

## 4. Discussion

Our study is the first to demonstrate that subjects with nodal HOA have reduced pain thresholds in their finger and wrist joints compared with non-HOA controls, suggesting that in chronic HOA people are sensitised to pain. Since non-DIP/PIP finger and wrist joints with low K-L scores for radiological severity also demonstrated reduced pain thresholds in our HOA group, our work suggests that participants with HOA have evidence of peripheral sensitisation. Our work is in agreement with other investigators who have demonstrated features of peripheral sensitisation in OA of the knee [7] and hip [21].

HOA is a chronic disease in which mechanisms of pain are not fully understood. Our study has shown that our cohort of HOA participants has significant functional impairment with mean HAQ scores of 0.8 which are similar to patients with established RA [22] and erosive HOA [23]. The significant functional impairment in our group was associated with evidence of hyperalgesia globally in finger and wrist joints (in participants with mainly DIP and PIP joint disease), demonstrating reduced pain thresholds across all finger and hand joints by algometer testing. Although we observed a strong correlation between increasing radiological severity and lower pain threshold in the PIP and DIP joints, globally reduced pain thresholds were observed in the hand including thumb and MCP joints that did not correlate with radiological severity. Our data also agrees with previous observations of lowered pain thresholds and hyperalgesia to mechanical stimuli in OA joints [24].

Limitations of our study include that we analysed a relatively small number of participants, that is, *n* = 26 in total. The HOA participants in this study were recruited from a specialist rheumatology clinic and therefore were likely to have more severe hand OA since they had been referred by primary care. The majority of our participants were also taking oral analgesics and could therefore have altered algometer pain thresholds changes as a result. Nevertheless, our data demonstrates how algometers could be useful biomarkers in OA pain studies to assess peripheral sensitisation and potential response to therapies. Recently, a rodent model of OA showed that treatment with NSAIDs only showed transient analgesic effects, whereas the effects of centrally-acting analgesics, including amitriptyline and gabapentin, were more sustained [25]. Of note, none of the participants in our study were on centrally acting analgesics or disease-modifying therapy, as demonstrated in Table 2. It is conceivable that future treatments for pain should include centrally acting agents for chronic conditions such as hand OA where no disease-modifying therapies exist. 

In conclusion, our study has found that patients with HOA not only have low pain thresholds in the most severely affected finger joints, that is, DIP/PIP joints, but also have reduced pain thresholds globally across all the finger and wrist joints tested. Higher functional impairment and anxiety were also experienced by our HOA participants compared with controls, suggesting that people with HOA may be sensitised to pain in the chronic state. Such considerations should be taken into account when assessing pain in HOA in clinical studies and in exploring novel therapeutic options for pain in HOA.

## Figures and Tables

**Table 1:** Group demographics. The age (mean ± SD), family history of OA, comorbidity, and information on previous joint replacement surgery in the hand OA and control groups are shown.

Characteristic	OA group Mean ± SD	Control group Mean ± SD	*P* value
Age	61.0 ± 13.7	52.8 ± 5.3	*P* = 0.12
Family history of OA	11/13	2/13	—
	Knee: 7/13		
	Foot/ankle: 5/13		
OA involvement in other joints	Spine: 1/13	—	—
	Shoulder: 1/13		
	Hip: 1/13		
Comorbidity			
(1) Hypertension	2/13	2/13	
(2) Hypothyroidism	0/13	2/13	
(3) Breast cancer	2/13	0/13	—
(4) Dyspepsia	1/13	1/13	
(5) Intervertebral disc herniation	0/13	1/13	
Previous surgery	Total hip replacement: 1/13	—	—

**Table 2:** Participant-reported outcome measures (PROMs). Data for duration of diagnosis, VAS in the hand OA versus the control group, Hospital Anxiety and Depression Scale (HADs) with subscales for anxiety and depression, and Health Assessment Questionnaire (HAQ) scores are shown with mean ± SD. *P* values are shown for group comparisons between hand OA and healthy control participants. Differences between groups were analysed using the Mann-Whitney *U*-test with significance considered at *P* ≤ 0.05.

Demographic	OA group	Normal group	*P* value
Duration of diagnosis (y)	6.1 ± 1.19	n/a	n/a
VAS score (mm) mean	59.3 ± 8.19	4.0 ± 1.89	<0.0001*
HADS depression score	3.2 ± 2.34	1.5 ± 1.66	0.05*
HADS anxiety score	7.5 ± 3.93	3.7 ± 3.20	0.01*
HAQ score	0.8 ± 0.10	0.11 ± 0.08	<0.0001*

Statistically significant values are marked on the table with*.

**Table 3:** Participant cohort demographics. The age, gender, family history of OA, past medical history and concomitant medication in the hand OA (OA1–OA13), and healthy controls (C1–C13) are shown.

Participant	Age	Sex	Occupation	PMH	Other nonanalgesic medication	Handed (R or L)	FH of OA	Duration of OA diagnosis (years)
OA1	57	F	Teacher	Carcinoma breast	Tamoxifen	R	Y	5
OA2	87	F	Retired clerical worker	Carcinoma breast	Aromatase inhibitor	R	Y	10
OA3	42	F	Cleaner	None	—	R	Y	1
OA4	53	F	Teacher	None	—	R	Y	3
OA5	75	F	Retired	None	—	R	Y	4
OA6	70	F	Lecturer	Hypertension	Antihypertensive	R	Y	10
OA7	49	F	Legal Assistant	None	—	R	N	5
OA8	67	F	Writer	Dyspepsia	Omeprazole	R	N	1
OA9	54	F	Management position	None	—	R	Y	8
OA10	71	F	Retired teacher	None	—	R	Y	15
OA11	41	F	Retired teaching assistant	None	—	R	Y	5
OA12	71	F	Retired librarian	Hypertension	Antihypertensive	R	Y	10
OA13	56	F	Special needs worker	None	—	R	Y	3
C1	52	F	Manager	Hypertension Hypothyroidism	Antihypertensive Thyroxine	R	Y	0
C2	47	F	Nurse	None	—	R	N	0
C3	63	F	Clerical worker	None	—	R	N	0
C4	52	F	Support worker	None	—	R	N	0
C5	55	F	Nurse	Hypothyroidism	Thyroxine	R	Y	0
C6	46	F	Secretary	None	—	R	N	0
C7	56	F	Receptionist	None	—	R	N	0
C8	51	F	Clerical worker	None	—	R	N	0
C9	55	F	Secretary	Hypertension Dyspepsia	Antihypertensive Lansoprazole	R	N	0
C10	50	F	Support worker	None	—	R	N	0
C11	57	F	Nurse	Hypothyroidism	Thyroxine	R	N	0
C12	58	F	Nurse	Disc protrusion	Paracetamol as required	R	N	0
C13	44	F	Nurse	None	—	R	N	0

PMH: past medical history, FH: family history.
